# “It's exactly what's needed”: a call to action for breastmilk studies of vertical HIV transmission in high-income countries

**DOI:** 10.3389/frph.2026.1902552

**Published:** 2026-07-22

**Authors:** Nell Freeman-Romilly, Bakita Kasadha, Shema Tariq, Angelina Namiba, Farai Nyatsanza, Nick Phillips, Tanvi Rai

**Affiliations:** 1 Oxfordshire Sexual Health Service, Oxford University Hospitals NHS Foundation Trust, Oxford, United Kingdom; 2 Nuffield Department of Primary Care Health Sciences, University of Oxford, Oxford, United Kingdom; 3 Institute for Global Health, University College London, London, United Kingdom; 4 4M Net Mentor Mothers CIC, London, United Kingdom; 5 Integrated Contraception and Sexual Health (iCaSH) Service Cambridgeshire, Cambridge, United Kingdom

**Keywords:** breastfeeding, breastmilk, HIV, infant feeding, lactation, postnatal, transmission, vertical

## Abstract

Increasing numbers of parents living with HIV in England and the United Kingdom choose to breastfeed their infants, yet guidelines for high-income countries (HICs) still recommend exclusive formula feeding to eliminate all risk of vertical transmission. The risk of HIV transmission through breastmilk is less than 1% when the feeding parent is virally suppressed on antiretroviral therapy. However, this data comes from studies conducted in low- and middle-income countries limiting their generalisability to contexts where parents are more likely to be virally suppressed on long-term antiretroviral therapy with access to regular viral load monitoring. The risk of HIV transmission through breastfeeding in the context of *long-term pre-pregnancy HIV viral suppression* is largely understood through indirect observational studies in HICs, suggesting the risk of HIV transmission may be lower than currently understood. We may be providing parents living with HIV in high income countries with infant feeding guidance that does not adequately reflect their level of risk. In this perspective paper, we explore the reasons behind the paucity of large-scale microbiological lactational research in this population group. We also present brief findings from the NOURISH-UK study which explored experiences of infant feeding decisions among UK-based mothers living with HIV: our participants expressed frustration about the lack of evidence applicable to their situation and were universally enthusiastic about donating breastmilk samples to enable research to take place. We deliver a call to action to researchers, clinicians, research funders and clinical networks to explore the logistics and practicalities of how this work could be supported.

## Introduction

Over half the global population of people with HIV are women and an estimated 1.3million people with HIV become pregnant each year (1, 2). Vertical transmission rates are low because of routine antenatal HIV testing, antiretroviral therapy (ART) during and after pregnancy, appropriate management of delivery and the neonatal period—and in high income settings recommendations to avoid breastfeeding and use formula milk instead (3, 4). The vertical transmission rate of HIV in England fell from 2.9% in 2000 to 0.3% in 2021 (5). With consistent access to ART and appropriate healthcare, people with HIV have a similar life expectancy as someone without HIV (6). If a person with HIV is on effective ART and has an HIV viral load less than 200 copies/mL, they cannot pass HIV during sex, referred to as Undetectable = Untransmittable (U = U) (7, 8).

However, U = U does not apply to HIV transmission through breastmilk. Without ART, the risk of HIV transmission through breastmilk is estimated between 16% to 20% (9). When the feeding parent is virally suppressed on ART this risk is reduced to less than 1%, but it is not zero (10, 11). However, almost all data on the risk of HIV transmission through breastfeeding with ART come from studies conducted in low- and middle- income countries (LMICs) (11, 12). These data may not be generalisable to high-income countries (HICs) where healthcare is often more accessible and individuals may have been on long term suppressive ART prior to pregnancy with access to resistance testing, routine HIV viral load monitoring and potentially a wider range of treatment options. In the UK between 2017 and 2023, ∼90% of new mothers and birth parents with HIV were aware of their HIV diagnosis prior to pregnancy, 95% of whom were on ART prior to conception (13). In comparison, routinely collected data across three sub-Saharan African countries (January to December 2018) found only about half of pregnant women were aware of their HIV before pregnancy, although varying between countries and age groups (14).

The World Health Organization advises that “Mothers known to be HIV-infected (and whose infants are HIV uninfected or of unknown HIV status) should exclusively breastfeed their infants for the first six months of life, introducing appropriate complementary foods thereafter, and continue breast feeding” (15). This is a harm reduction approach for when access to clean water and formula milk is inconsistent. In the UK and similar HICs, national HIV guidance continues to advise avoidance of breastfeeding completely stating “feeding your baby with formula milk in a bottle is the only way to make sure the risk of passing on HIV to your baby is zero” (16). Guidance for parents with HIV living in HICs generally assumes consistent access to safe water and infant formula. It is based on a combination of the LMIC-based research demonstrating residual transmission risk from breastfeeding in the context of lower infant morbidity and mortality secondary to gastrointestinal and respiratory infections—making formula feeding the ideal way to avoid all risk of HIV transmission (9). This discrepancy in advice, however, causes parents anguish and confusion (17).

These guidelines have changed with increasing appreciation of why mothers and parents with HIV may want to breastfeed, moving away from advising *strict* avoidance of breastfeeding (18) towards a person-centred, harm-reduction approach that can enable “supported breastfeeding” under some circumstances (19).

**Figure 1:**
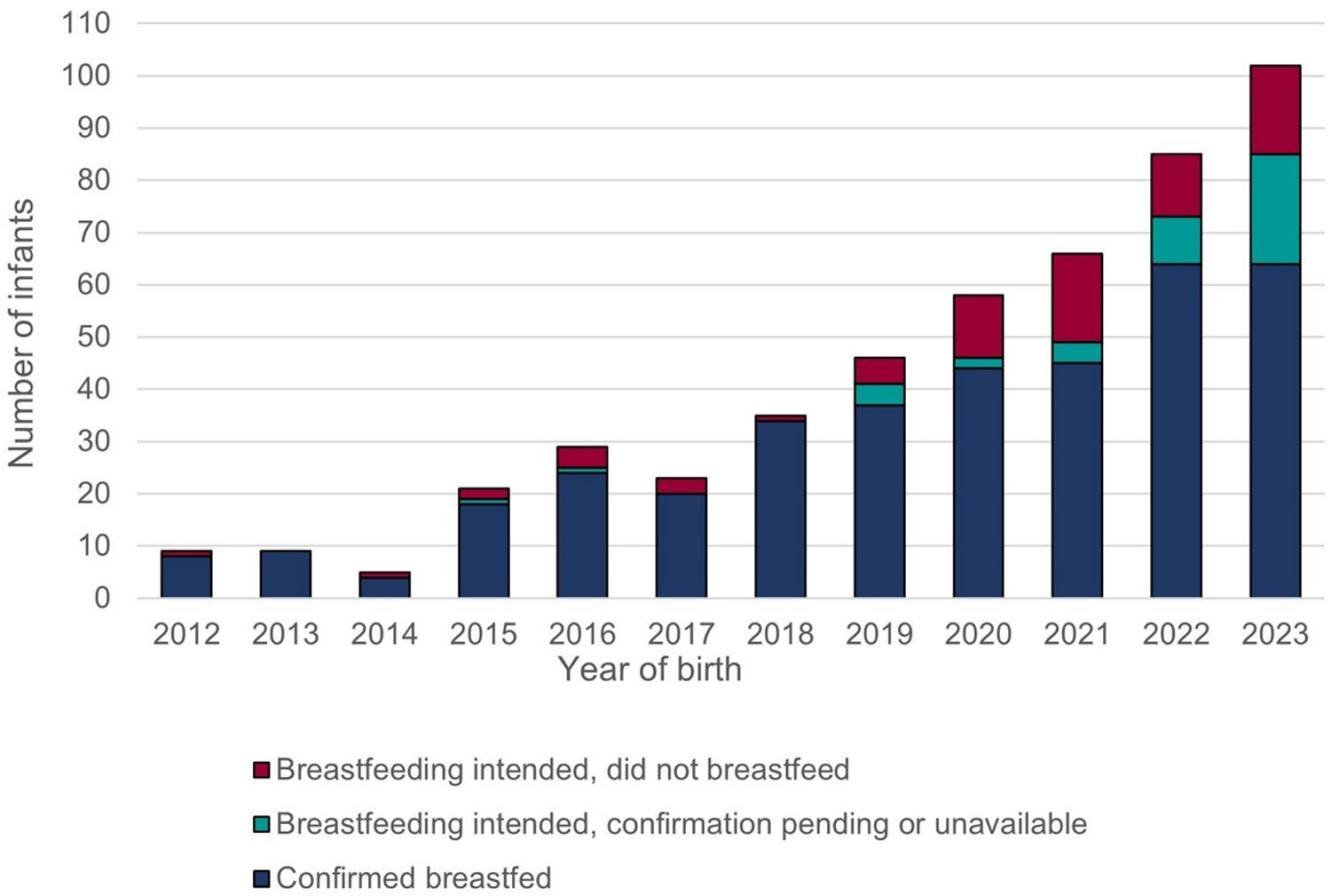
Reports of intended and/or confirmed supported breastfeeding in England, 2012–2023 (20).

There is growing interest in breastfeeding among women and birthing parents with HIV (13). In the UK the number of individuals with HIV who reported an intention to breastfeed rose from <10 in 2012 to >100 in 2023, with a corresponding increase in numbers of individuals reporting breastfeeding (Figure 1) (13).

In HICs, people with HIV describe multiple reasons for wanting to breastfeed including bonding with their infant, the health benefits of breastmilk, and a desire to avoid stigma as a result of not breastfeeding, a recommendation that is further amplified in diasporic communities where not breastfeeding can function as a surrogate marker for having HIV (21–25). Adherence to formula feeding is impacted by a lack of consistent provision of free formula and sterilising equipment to parents with HIV, however, in December 2025 the UK government has promised to provide free formula milk for infants of all women and parents living with HIV (17, 21, 22, 24–28). Parents living with HIV in HICs thus find themselves caught between multiple, often conflicting health and social imperatives regarding infant feeding, and increasingly nuanced guidelines that still generally advise against breastfeeding (17, 19, 22, 23, 29).

There have been no reported cases of vertical HIV transmission in the emerging data from HICs of parents with HIV who self-reported breastfeeding while being virally suppressed (30–32). One study which did review HIV reservoir in breastmilk included one person with HIV who was virally suppressed and on long-term ART. No HIV RNA, and no evidence of potentially replication-competent HIV was identified in their breastmilk fluid or cells (33). While these results are encouraging, large scale, non-observational studies investigating the relationship between viral suppression in the long-term virally suppressed feeding parent and vertical HIV transmission remain absent.

It is well established that the plasma HIV viral load of the feeding parent remains the largest determinant of HIV transmission through breastmilk (34). However, the incomplete understanding of the specific mechanisms of HIV transmission in the context of long-term viral suppression means there are limited specific interventions to lower the risk of breastmilk transmission. Existing interventions are currently centred on reinforcing consistent ART adherence and avoidance of breastfeeding either completely or during times of potential risk (e.g., mastitis) (19, 34).

Dugdale et al.'s 2025 review on the effect of maternal viral load on perinatal and postnatal HIV transmission explain this data gap, “There were considerably fewer studies that provided data on postnatal vs. perinatal transmission risks, in part because settings with the greatest access to serial mHVL [maternal HIV Viral Load] monitoring are also those in which breastfeeding among women with HIV was discouraged until 2018 or later.” (35). Research agendas may be affected by biases within institutions, funding bodies and academic cultures in HICs, influencing both who is represented in research and which topics are prioritised (36, 37). More than 75% of pregnancies in people with HIV in England are in women of Black or other racially minoritised ethnicity (13). Misogynoir, describing the intersecting effects of racism and misogyny experienced by Black women, is understood as influencing inequities within health research agendas, including funding priorities, epistemic authority, and whose voices are valued (38). Assumptions remain among research communities and their academic and clinical institutions about how certain groups and populations are unlikely to participate in research—that they can be “hard to reach”—and unequal health research agendas may further inhibit investigation in this field (39–42). Additionally, UK HIV research has historically focused more on men who have sex with men (MSM) populations than women (43, 44) and inclusive meaningful involvement of women throughout research is still needed (45).

### Women's views on breastmilk studies in the UK

NOURISH-UK was a qualitative study about infant feeding decisions among people living with HIV, comprising semi-structured interviews with 36 cisgender women living with HIV in the UK who were pregnant (*n* = 8) or had had a live birth in the preceding 12-months (*n* = 28) and 27 women were of Black or other racially-minoritised ethnicity. Our study methods, analyses and major findings are presented in detail elsewhere (17, 21, 22, 27).

Participants prioritised safeguarding the health of their infants, which for them meant minimising the risk of vertical transmission whilst also considering the benefits of breastmilk. Many judged the risk to be low enough to consider the emotional, social, and biological benefits of breastfeeding, but the absence of the reassurance from personally relevant large-scale research, equivalent to the large studies conducted in LMICs, remained a persistent and overwhelming source of concern. They wanted detailed information that was applicable to their setting, and guidance on how to reduce the risk of vertical transmission if they did decide to breastfeed (22). On being informed of the lack of research in this area [see Box 1], all women interviewed expressed a willingness to participate in breastfeeding studies. All women chose pseudonyms which are included with their quotes below.

Participants voiced frustration with the incomplete and ambiguous information provided to them to support what they felt was a profoundly important decision:

“There's a deficit of data, it's harder to make more informed decisions”

(Kay, 31, White Other, pregnant, planning to breastfeed)^1^

Others commented on the lack of contemporary data that was relevant to their lives in the UK and questioned why data on HIV transmission through breastfeeding seemingly lagged behind studies that underpinned U = U (7, 8).

“…it was studies in Africa that was done, you know, twenty years ago and I’m like yeah, I can’t relate to that, you know.”

(Amina, 23, British Asian, pregnant, undecided regarding feeding)

“It's exactly what's needed because, if now undetectable equals untransmissible then it's very likely that that it's the same for breastmilk, I would think”

(Tina, 36, White British with twins 7.5 months old, formula feeding)

Some participants initially expressed concern that providing breastmilk samples might reduce the amount available for their infant. However, on learning how small the sample would be, approximately 10 mL or a tablespoon, and seeing the collection tube, these concerns were alleviated. Many expressed a preference for flexibility in collection methods. Some preferred the convenience of home sampling with courier collection, particularly in the early postnatal period when leaving the house may be difficult:

For me [um] because [as] a new mum you don't wanna go out anywhere […] so for me if somebody can collect it at home would be better.

(April, 40, Black African with a 4 month old, formula feeding)

Others preferred to provide samples during routine postnatal clinical appointments, citing greater efficiency, more robust sample collection, and increased privacy; especially when their HIV status has not been shared to other household members.

Yeah, that [providing samples during clinical appointments] would be best, yes, because you go there and then it's you alone that you go there. You do it with the professional so it's safe, it's much safer, safer.

(Emily, 41, Black African with 5-month old, formula feeding)

Concerns about privacy and practicalities were also raised in relation to home collection or postal returns:

“For me my mum might be, ‘Why the f*** is someone at my door for the breastmilk?’ Or I don’t know, post, could you like post it, that kind of thing? But then again, if I’m post-partum I’m not going to go [to] the post box but if it were like once a month or something, yeah, why not.”

(Amina, 23, British Asian, pregnant, undecided regarding feeding)

Overall, donating breastmilk samples for research was viewed as highly acceptable to study participants. They expressed strong altruistic motivations, particularly in contributing to research relevant to future parents living with HIV in their setting:

“I’d say yes because it's to help others as well isn’t it? It takes one person to decide to donate, otherwise nothing can move forward”

(Camille, 44, Black African with 9-month old baby, formula feeding after experiencing little support to breastfeed)

Even in the absence of direct personal benefit, participants described a keenness to help others:

“I would do that knowing that it's going to be beneficial to other kids and other parents, I would do that”.

(Emily, 41, Black African with 5-month old, formula feeding)

### Our call to action: undertaking breastmilk studies with people living with HIV in high income countries

A growing number of people living with HIV in the UK and other HICs wish to breastfeed but there is an absence of data on postnatal transmission of HIV through breastmilk in the context of long-term effective maternal viral suppression enabled through sustained access and adherence to ART (20, 46, 47). The consequence is national guidance, developed in the absence of evidence generated in a fully comparable setting, which advises potentially unnecessary avoidance of breastfeeding.

Breastfeeding is globally promoted as one of the most effective ways to give babies the healthiest start in life (48) and “breast is best” campaigns are omnipresent in antenatal and postnatal clinical and community spaces all over the UK and other countries (49). In addition to reducing infection and nutrition risks in infancy, breastfeeding supports the child's neurological development and reduces the risk of future obesity and chronic disease (50). It supports child spacing and provides the feeding parent with protection against type 2 diabetes, cardiovascular disease, breast and ovarian cancers (51). Restricting breastfeeding may be indirectly harmful by preventing the baby and feeding parent from accessing its short and long-term benefits. More than a third of women with HIV in England who were pregnant between 2022 and 2023 reported to their healthcare provider that they had complex social circumstances (13) and half of our NOURISH-UK sample reported struggling to afford basic needs all or some of the time (22). While the 2025 HIV Action Plan for England endorses funding, “formula milk and sterilising equipment provision for infants born to women living with HIV”, guidance advising formula feeding as the safest option in the context of maternal HIV may inadvertently be limiting access to the benefits of breastfeeding for the very infants who might benefit most (52).

### Priorities

In settings with reliable access to ART and monitoring, HIV is often considered a clinically manageable chronic disease (53). Despite the persistent burden of HIV-related stigma and morbidity that accompanies living with HIV, addressing the HIV epidemic undeniably lacks the urgency and resourcing that drove much of the innovation of the late 1990s and 2000s (54–57).

NOURISH-UK findings highlight that infant-feeding decisions are often experienced as highly-charged, complex and emotionally gruelling, particularly among Black or other racially minoritised participants from cultures where breastfeeding is the norm (17). This is an important distinction, as breastfeeding levels among the general population in HICs are often low (58). In the UK, more than half of mothers from the majority white ethnic group did not breastfeed at all, however this was the case for only 15% of Black or Black British mothers (59).

While formula feeding removes all HIV transmission risk, it also takes away the social and biological short-term and long-term benefits of breastfeeding (60). NOURISH-UK participants described additional social risks associated with formula feeding, including stigma, judgement and the fear that their HIV status would become known (17, 21, 22, 27) similar to findings from other high-income settings (61–65). Framing formula feeding as a *straightforward* “risk elimination” strategy elides the broader social and structural contexts in which infant feeding decisions are situated and is symptomatic of the racialised biases that fail to recognise the many forms of personal risk that Black and minoritised people are forced to navigate in HICs.

### People

Our NOURISH-UK findings demonstrate high acceptability and desire for breastmilk studies in high-income settings among new mothers with HIV, adding to existing evidence (25). As perinatal HIV guidelines become more supportive towards breastfeeding, the proportion, and number, of parents with HIV in HICs like the UK who choose to breastfeed is likely to continue to increase (19, 20). However, any study on this topic would need to include multiple sites and stay open for several years to reach adequate power for statistical analysis. International consortia such as European Pregnancy and Paediatric Infections Cohort Collaboration provide examples of how this can be overcome by collaborating across several countries and provide a template for a similar HIV and breastmilk research collaboration (66). One consideration might be that inclusion criteria could be centred on person centred biomedical criteria, such as, living with HIV and on long-term pre-pregnancy viral suppression with regular viral load monitoring prior to pregnancy, rather than the Gross National Income category of their country of residence (67). Including long-term virally supressed study participants from Upper-Middle Income economies with well-established HIV testing and care and where there is routine viral monitoring would maintain study validity and support robust analyses.

### Practicalities

#### Technical considerations

Breastmilk is a biofluid with a highly variable composition; it would be important to consider what type of milk is being collected; for example, foremilk, midmilk, hindmilk, or complete milk from full breast emptying (68). Milk can be processed as either whole milk, milk fat layer or the remaining supernatant once the lipid layer is removed. These would need to be standardised amongst donation samples, with the complexity and volume of collection balanced between scientific rigour and what is acceptable to and practical for study participants.

Viral load assay would likely involve quantification of HIV RNA by Reverse transcription-quantitative polymerase chain reaction (RT-qPCR), requiring good quality RNA. This can be achieved by collecting milk into a tube containing a preservative such as RNAlater, but consistent sample collection, transport and storage conditions and preservation medium would need to be standardised between potentially multinational collection sites.

Finally, commercial HIV viral load assays are validated solely for EDTA plasma, and occasionally serum, and not intended for human breastmilk specimens (69–72). Assays can be validated for research study purposes, however this might necessitate centralised breastmilk sample testing.

Viral load testing of breastmilk as part of a research study would not be able to provide clinically applicable advice to a breastfeeding parent about whether it would be “safe to breastfeed”. While breastmilk viral load testing is achievable, the information it would yield in a research study would not be validated as a proxy measure of blood viral load testing and would not replace the recommended monthly review for HIV viral load venepuncture burden associated with current infant feeding guidance for those who breastfeed (19). This might eventually change following careful review of the data, however, would need to be clearly communicated to potential study participants and may impact recruitment and study retention. The additional logistical demands of participating in a research study during the already challenging postnatal period may understandably deter people from enrolling. However, those who breastfeed already have additional clinical monitoring compared with those who formula feed, so even separate from the NOURISH-UK findings on patient willingness, there may be greater tolerance for engagement with healthcare among this population (19).

### Opportunities for future research

There is a clear need for future research centred on informing infant feeding interventions for parents living with HIV with long term viral suppression. Examples of these questions are in Box 1.

Box 1 **Examples of research questions that might inform targeted infant feeding interventions for parents living with HIV with long term viral suppression**

- What is the relationship between viral load in breastmilk and plasma HIV viral load among individuals with sustained viral suppression?
- How does the breastmilk viral load vary over a 24 h cycle and over the course of breastfeeding from colostrum to transitional milk to mature milk and then over the next weeks and months?
- How does breastmilk viral load change between the different phases– foremilk, midmilk, and hindmilk?
- How is breastmilk HIV viral load affected by common breastfeeding conditions such as mastitis and cracked nipples, and what is the time course for changes to breastmilk viral load?
- What is the impact of co-existing short- and long-term health conditions in the feeding parent and infant, e.g., CMV, on breastmilk viral load and the risk of vertical transmission?
- Does testing breastmilk HIV viral load provide clinically helpful information that supports patient care?
- What is the duration of viral suppression needed to breastfeed with minimal risk of HIV transmission?
- What are the risks that define whether ART for the infant should continue for the duration of breastfeeding and does breastmilk HIV viral load analysis have a role in determining this?
- What is the impact of long-acting HIV treatment on breastmilk viral load? Does this change in the time between injections even when plasma viral load is stable?

Future research on postnatal vertical transmission in breastfeeding parents with HIV could use existing international collaborations on multiple neonatal health conditions to address the comparatively small numbers of eligible populations within countries. An additional approach might be to use data collected through routine care, complemented by nested sub-studies involving breastmilk collection and analysis. Using standardised open access data formats, for example HIV Cohorts Data Exchange Protocol and Observational Medical Outcomes Partnership Common Data Model would allow analyses of pooled data collected across different sites. Some collected data may already exist and “just” needs to be identified and repurposed for secondary analysis.

## Conclusion

Technological constraints, recruitment challenges, limited site capacity, and funding barriers are challenges that are likely to persist, especially in a limited global health funding landscape. There remains, however, a clinical and scientific imperative to address the questions that matter most to expectant and new parents with HIV. Our NOURISH-UK participants expressed a need for relevant, evidence-based guidance on HIV and infant feeding. Their willingness to contribute biological samples underscores the acceptability and feasibility of research in this area. As the number of virally suppressed parents living with HIV who wish to breastfeed continues to grow, important questions remain unanswered. Addressing these will require a sustained, collaborative effort and a commitment to centring the experiences and priorities of people living with HIV in shaping the future of research into HIV and infant feeding.

## Data Availability

The original contributions presented in the study are included in the article. Further inquiries can be directed to the corresponding author.

## Footnotes

^1^Reported quotes are accompanied by participant demographics with pseudonyms.
